# The Efficacy and Safety of Leflunomide for the Treatment of Lupus Nephritis in Chinese Patients: Systematic Review and Meta-Analysis

**DOI:** 10.1371/journal.pone.0144548

**Published:** 2015-12-15

**Authors:** Heng Cao, Yuefeng Rao, Lin Liu, Jin Lin, Hongyu Yang, Xingguo Zhang, Zhong Chen

**Affiliations:** 1 Department of Rheumatology, The First Affiliated Hospital, College of Medicine, Zhejiang University, Hangzhou, China; 2 Department of Pharmacy, The First Affiliated Hospital, College of Medicine, Zhejiang University, Hangzhou, China; 3 Department of Pharmacology, College of Pharmaceutical Sciences, Zhejiang University, Hangzhou, China; Nippon Medical School Graduate School of Medicine, JAPAN

## Abstract

**Objective:**

To evaluate the clinical efficacy and safety of leflunomide as a new immunosuppressive medicine in lupus nephritis (LN) through a meta-analysis.

**Methods:**

A systematic review evaluating the efficacy and safety of leflunomide compared with cyclophosphamide in adult patients with LN was performed. Data from relevant randomized controlled trials (RCTs) performed before December 2014 was collected from several databases (PubMed, Embase, Cochrane Library, CNKI and CBM). No language restrictions were applied. Efficacy outcomes included overall remission, SLE Disease Activity Index (SLEDAI) score, 24-hour proteinuria and serum creatinine. Safety data were analyzed. The effects of treatment on these outcomes were summarized as relative risks (RRs) with 95% confidence intervals (CIs) and mean differences were pooled using a fixed or random effects model.

**Results:**

Eleven RCTs with Jadad score of 3 or greater were identified and included a total of 254 patients. Cyclophosphamide was served as the control drug in all trials. The SLEDAI score, urine protein level and serum creatinine decreased significantly following leflunomide treatment (*P*<0.05). Leflunomide was superior to cyclophosphamide in achieving complete and total remission, but no difference in SLEDAI score was found between these two treatments (*P*>0.05). Additionally, patients receiving leflunomide treatment showed favorable renal function profiles, especially regarding the 24-hour proteinuria (mean difference: -0.58, 95%CI: -0.78~-0.37, *P*<0.01) and serum creatinine (mean difference: -0.20, 95%CI: -0.39~-0.01, *P*<0.05). In the safety comparison, leflunomide was safer than cyclophosphamide regarding adverse drug reactions (ADRs), including liver damage (RR = 0.53, 95%CI: 0.33~0.87, *P*<0.05), alopecia (RR = 0.38, 95%CI: 0.17~0.85, *P*<0.05), leukopenia (RR = 0.25, 95%CI: 0.08~0.77, *P*<0.05) and infection (RR = 0.54, 95%CI: 0.32~0.92, *P*<0.05), without increased risk of gastrointestinal reaction, rash or herpes zoster infection.

**Conclusions:**

Leflunomide is a promising therapy for LN treatment, primarily because of the comparable efficacy and favorable safety profile determined by this meta-analysis of RCTs. Larger RCTs with longer duration of observation are necessary to provide strong evidence of the efficacy and safety of leflunomide in LN patients.

## Introduction

Approximately 35% of adults have clinical evidence of nephritis when they were diagnosed with systemic lupus erythematosus (SLE), with more than half developing nephritis during the first 10 years of disease[1]. The prevalence of lupus nephritis (LN) is significantly higher in Asian, African American and Hispanic populations and is higher in men. LN is considered as one of the most common causes of mortality in patients with SLE[2].

Currently, the main immunosuppressive drugs for LN include cyclophosphamide, mycophenolate mofetil, and azathioprine. Unfortunately, many patients experience adverse drug reactions (ADRs) to these drugs, such as infection, liver damage, and leucopenia, which contribute to increased mortality. Therefore, there is an urgent need for new, more effective therapeutic methods with more favorable safety profiles[3,4].

Leflunomide is an isoxazole immunomodulatory agent that inhibits dihydroorotate dehydrogenase (an enzyme involved in pyrimidine synthesis) and leads to decreases in DNA and RNA synthesis and cell proliferation. Leflunomide has been confirmed to be as effective as methotrexate and sulfasalazine in the treatment of rheumatoid arthritis (RA)[5]. Since it was introduced in 1998 for RA treatment, leflunomide has been increasingly used in clinical applications[6]. Many experimental models and clinical studies have showed that leflunomide has an obvious ameliorative effect against SLE and LN. However, the efficacy and safety of leflunomide and the prognosis of patients treated with leflunomide remain under debate [7].

Meta-analyses are increasingly used to assess the clinical efficacy and safety of treatments, and the superiority of this analytical method is obvious when compared with other analytical methods. This review presents a meta-analysis of published prospective clinical trials to assess the efficacy and safety profile of leflunomide in the treatment of LN. Specifically, we attempted to determine whether this mechanism-specific immunosuppressive agent is equivalent to or more effective than the classic treatment of cyclophosphamide, and whether it may be preferable in certain cases.

## Materials and Methods

### Data sources

We conducted a systematic review and meta-analysis of randomized controlled trials (RCTs) that compared leflunomide to cyclophosphamide in adult patients with LN. This meta-analysis was conducted in accordance with PRISMA guidelines (S1 Table). Two independent reviewers (Dr. Rao and Dr. Cao) performed electronic searches of the following databases: PubMed, Embase, Cochrane Library, CNKI and CBM. The search terms included *‘Leflunomide’*, *‘LEF’*, *‘efficacy’*, *safety’*, *‘autoimmunity’*, *‘autoimmune’*, *‘systemic lupus erythematosus’ and ‘SLE’* and were entered as both medical subject heading (MeSH) terms and text words. Meeting abstracts were searched in the Web of Science. Boolean operators such as ‘‘AND” and ‘‘OR” were also used in the electronic search. No language restrictions were applied. The complete search strategy used to search Pubmed is described in S2 Table. All published RCTs that included patients treated with leflunomide for at least 24 weeks were included. The control intervention was either placebo or another conventional treatment. Studies of treatment protocols involving the co-administration of other investigational agents were also included. And a secondary search of references was performed to verify that no missing any important article.

### Inclusion and exclusion criteria

Studies were included if they met the following criteria: (1) examined leflunomide as an induction therapy for LN, (2) recorded the necessary data regarding therapeutic efficacy and safety, and (3) enrolled patients with a diagnosis of LN based on the ACR criteria. We excluded studies that included pediatric patients (≤16 years old). Two authors (Dr. Rao and Dr. Cao) independently evaluated the retrieved studies. All studies that did not meet the inclusion criteria, such as case series, reports and retrospective longitudinal studies were omitted.

### Outcome measures

Three clinical outcomes were studied: 1) remission rate (including complete remission-CR, partial remission-PR and total remission) and change in SLEDAI score; 2) renal function (24-hour proteinuria and serum creatinine); and 3) ADRs, including liver function abnormality, gastrointestinal reaction, rash, alopecia, leucopenia, infection, menoxenia and herpes zoster infection.

The CR criteria for LN were as follows: normal serum creatinine and serum albumin, inactive urinary sediment, and a 24-hour urinary protein level <0.5 g. The PR criterion of LN was ≥50% improvement in all renal parameters that were abnormal at baseline without deterioration in any parameter.[4]

### Quality evaluation

The methodological qualities of the included trials were assessed using the Jadad score, which judges descriptions of randomization, blinding, and dropouts/withdrawals from trials[8]. The Jadad scale ranges from 0 to 5 points, with a score ≤2 indicating low quality and a score ≥3 indicating high quality[9].

### Statistical analysis

Data were extracted and summarized as medians or means and SDs as provided by the authors. The indicators of heterogeneity between studies were analyzed to determine whether these indicators could be combined, and heterogeneity was analyzed using the χ2 test with N−1 degrees of freedom. A *P* value of 0.05 was regarded as the critical value for homogeneity. Continuous outcome data from individual trials were meta-analyzed using the weighted mean difference (WMD) as the combined effect. If the studies included were homogeneous, they were meta-analyzed using the fixed effects model to estimate the combined effect. If the studies included were heterogeneous, they were analyzed using the random effects model to estimate the combined effect. When the confidence intervals did not intersect at zero, the difference in treatments was statistically significant at the 0.05 level. All statistical analyses were performed using Review Manager 5.2 statistical software.

Risk ratios were chosen for effect measures using the inverse variance model with 95% confidence intervals (random-effects model) for dichotomous variables. Statistical heterogeneity among the results was assessed using the I^2^ statistic, which is easily interpreted. The I^2^ values are measured as a percentage that ranges from 0 to 100%, and heterogeneity can be classified as low (I^2^<25%), moderate (25%<I^2^<50%), or high (I^2^>50%) [10]. Publication bias was explored using funnel plots and Egger test.

## Results

**Fig 1:**
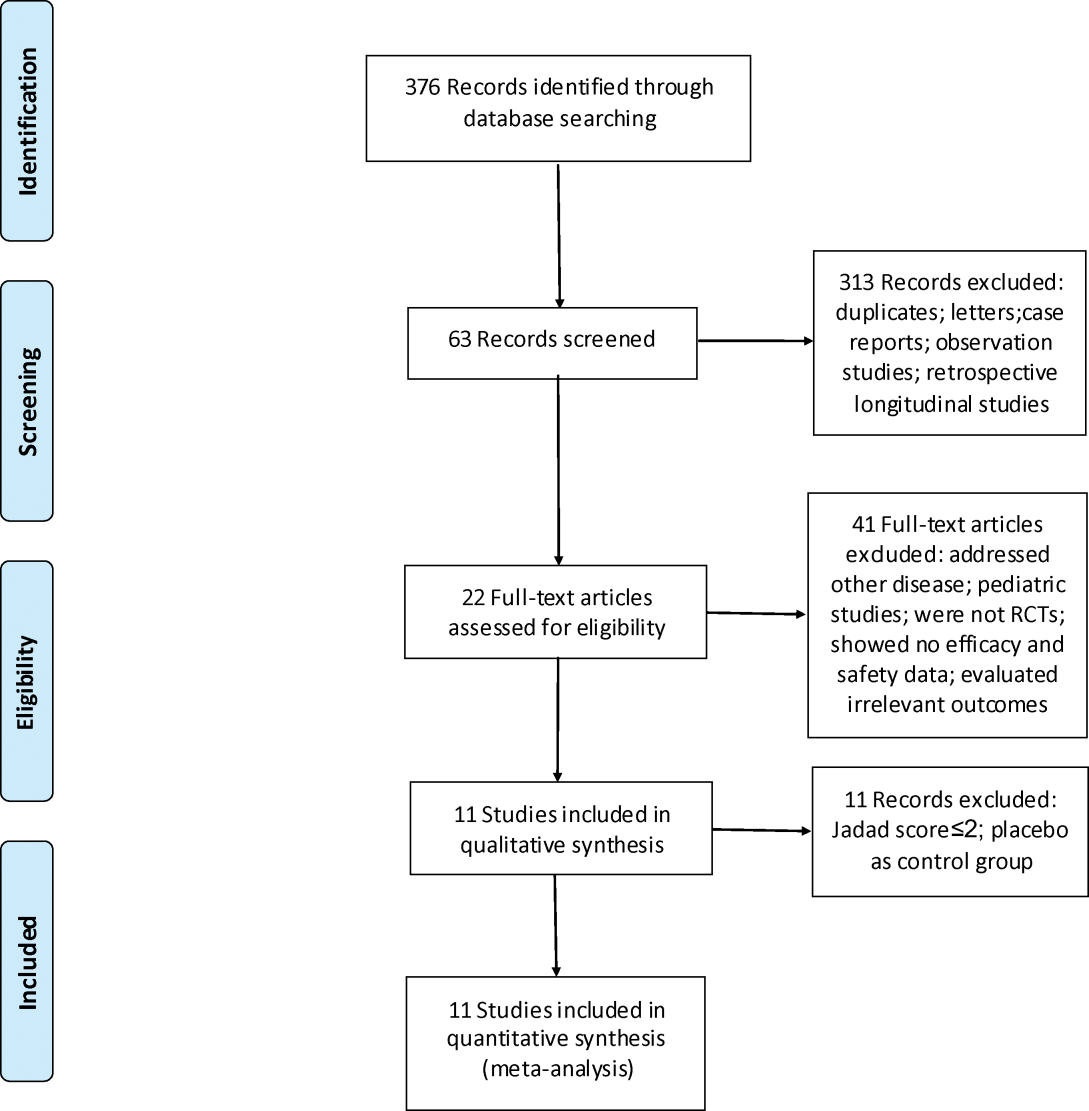
PRISMA 2009 Flowchart depicting the selection process for the studies included in the meta-analysis.

Our systematic review of electronic databases identified 376 relevant articles (75 from PubMed, 121 from Embase, 9 from Cochrane Library, and 171 from CNKI and CBM) (Fig 1). After title and abstract screening, 63 articles were retrieved for full-text review, and 22 eligible RCTs were identified. Eleven studies (S3 Table) were excluded due to low quality (less than 3 points on the Jadad’s scale). At the end of screening, 11 studies were selected for the meta-analysis, as summarized in Table 1[10–20]. All studies provided data regarding the comparison of the efficacy of leflunomide with that of cyclophosphamide. The approximate dosages used were as follows: oral prednisone, 0.5 to 1.0 mg/kg/day; oral leflunomide, 20 to 50 mg/day; intravenous cyclophosphamide, 0.5 to 1.0 g/m^2^ body surface area once per month. The follow-up period ranged from 6 to 12 months.

**Table 1:** Summary of available information for each study included in the analysis.

Author (study)	No. of patients		LEF(po)	CTX(iv)	Age years	women%	Outcome	CR [N(%)]		PR [N(%)]		Follow-up duration	Jadad's score
	LEF	CYC			LEF/CYC	LEF/CYC		LEF	CYC	LEF	CYC		
**Chen 2003**	12	20	50mg/d*3d→20mg/d	0.6g/2w	32/31	92/90	proteinuria, SLEDAI, ALB, ADR, SCr	NR	NR	NR	NR	6 months	3
**Cao 2007**	17	18	50mg/d*3d→30mg/d	1.0g/4w	34/41	86/91	CR, PR, proteinuria, SLEDAI, ALB, C3, SCr	9(52.9)	8(44.4)	6(35.3)	5(27.8)	6 months	3
**Li 2007**	21	18	100mg/d*3d→20mg/d	0.6g/2w	NR	NR	CR, PR, NR, proteinuria, SLEDAI, ANA, anti-dsDNA, ALB, ADR	7(33.3)	6(33.3)	8(38.1)	5(27.8)	6 months	3
**Wu 2008**	18	20	50mg/d*3d→30mg/d	1.0g/4w	35/34	83/80	CR, PR, NR, proteinuria, SLEDAI, ALB, SCr, C3, ANA, anti-dsDNA, ADR	6(33.3)	6(30.0)	8(44.4)	9(45.0)	12 months	3
**Chen 2010**	19	18	100mg/d*3d→20mg/d	0.6g/2w	NR	NR	CR, PR, NR, ADR	6(31.6)	5(27.8)	8(42.1)	7(38.9)	6 months	3
**Mo 2010**	31	31	50mg/d*3d→20mg/d	1.0g/2w	32/32	100/100	CR, PR, SLEDAI, proteinuria, SCr, ALB,WBC, ADR	5(16.1)	2(6.5)	22(71.0)	20(64.5)	6 months	4
**Pan 2010**	34	34	60mg/d*3d→30mg/d	1.0g/4w	31/30	85/88	CR, PR, NR, proteinuria, SCr, ALB, C3, anti-dsDNA, ADR	11(32.4)	9(26.5)	18(52.9)	18(52.9)	12 months	4
**Dong 2011**	20	20	50mg/d*3d→30mg/d	1.0g/4w	NR	NR	proteinuria, SCr, C3, ALB, ADR	NR	NR	NR	NR	6 months	3
**Peng 2011**	42	42	50mg/d*3d→40mg/d	1.0g/4w	29/29	93/90	CR, PR, NR, proteinuria, SCr, CRP, ESR, ADR	26(61.9)	16(38.1)	11(26.2)	15(35.7)	6 months	4
**Xia 2012**	21	21	20mg/d*3d→10mg/d	1.0g/4w	NR	NR	CR, PR, NR, proteinuria, SCr, CRP, ESR, ADR	13(61.9)	7(33.3)	6(28.6)	6(28.6)	6 months	3
**Zhu 2013**	19	18	20mg/d	1.0g/4w	33/35	84/83	CR, PR, NR, proteinuria, SCr, CRP, ESR, ADR	8(42.1)	5(27.8)	9(47.4)	8(44.4)	6 months	3

Abbreviations: LEF, leflunomide; CYC, cyclophosphamide; Pred, prednisone; PO, per os; IV, intraveno; SLEDAI, SLE Disease Activity Index; ALB, albumin; ADRs, adverse drug reactions; SCr, serum creatinine; WBC,white blood cell count; CR, complete remission; PR, partial remission; NR, no remission; C3, complement 3; ANA, antinuclear antibodies; CRP, C reactive protein; ESR, Erythrocyte Sedimentation Rate; NR, not reported.

**Fig 2:**
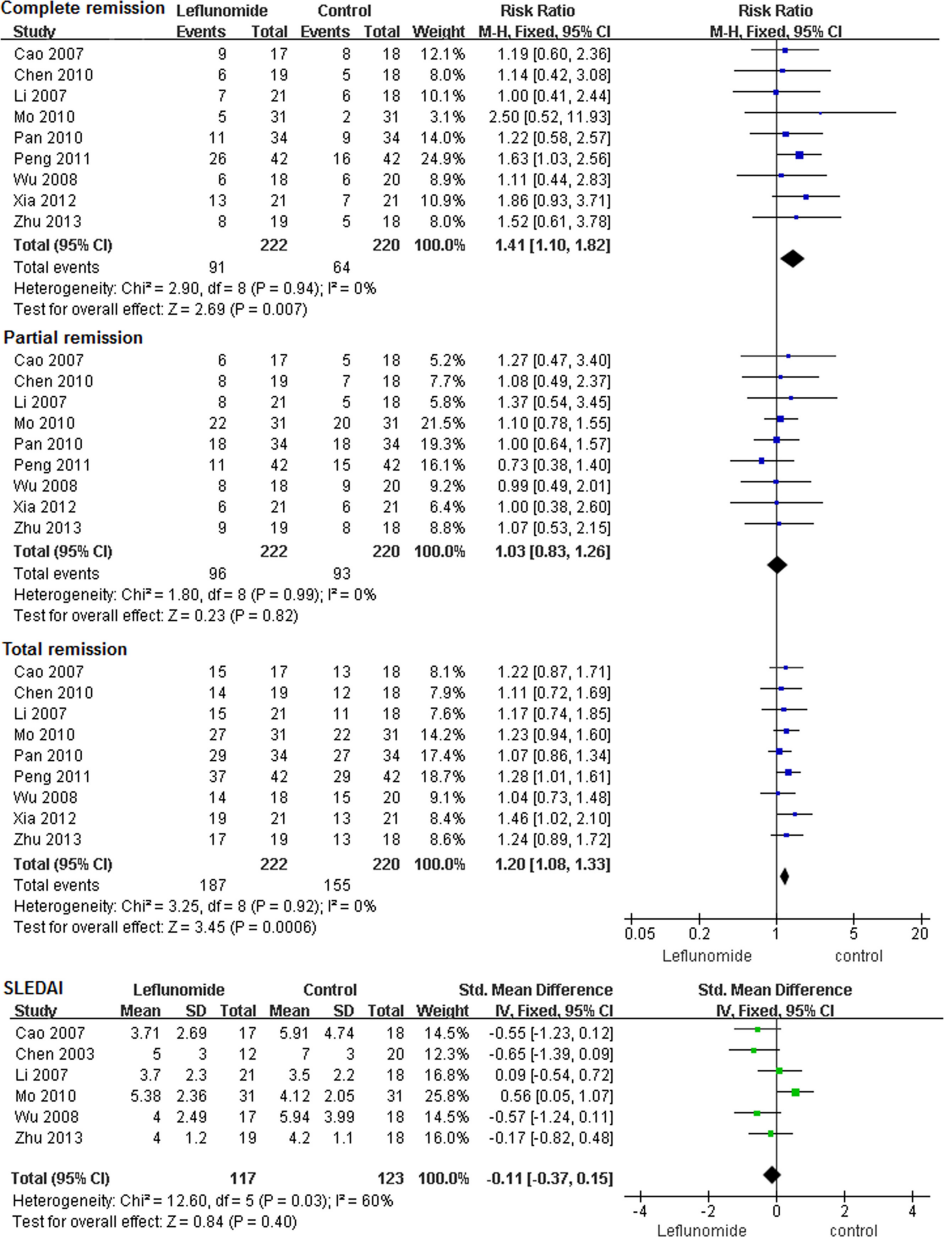
Results of the meta-analysis of remission and SLEDAI score in LN patients treated with leflunomide.

The relative risk differences for the oral leflunomide group compared to the cyclophosphamide group are shown in Fig 2. Nine studies analyzed clinical remission as an outcome, and leflunomide showed a total remission (RR) of 1.20 (*P*<0.01, 95%CI 1.08~1.33) and an I^2^ of 0%. CR and PR were 1.41 (*P*<0.01, 95%CI 1.10~1.82) and 1.03 (*P*>0.05, 95%CI 0.89~1.26), respectively. No significant heterogeneity was observed for the outcome of remission. Six studies analyzed the SLEDAI score as an outcome, and overall, it was not significantly different between groups (RR = -0.11, 95%CI = -0.37~0.15, *P*>0.05) but present high heterogeneity (*P* < 0.05, I^2^ = 60%). This result indicated that treatment with leflunomide had similar effects on the SLEDAI score compared with cyclophosphamide.

**Fig 3:**
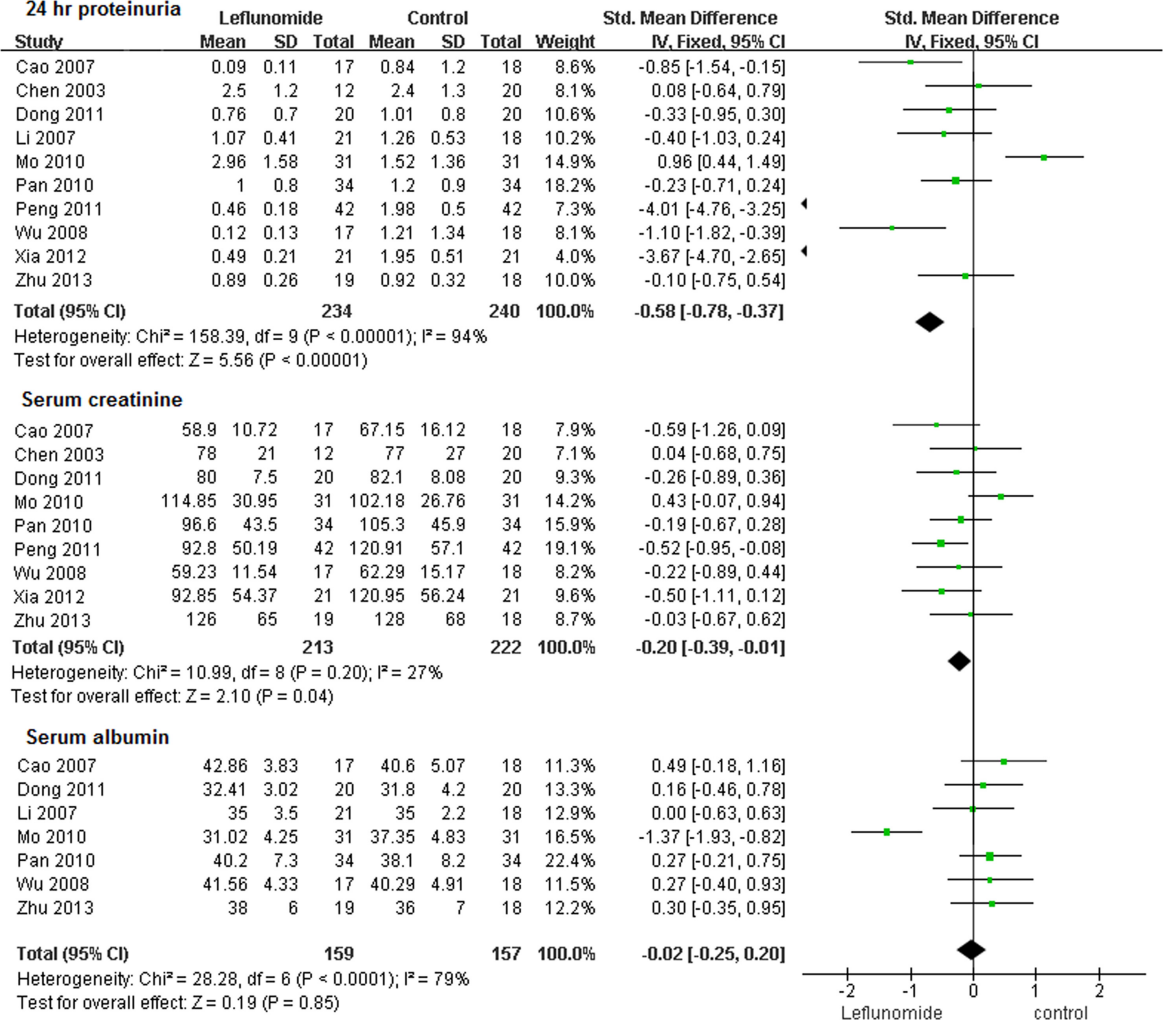
Results of meta-analysis of renal function in LN patients treated with leflunomide.

Twenty-four hour proteinuria and serum creatinine were chosen to evaluate renal function because these outcomes showed apparent uniformity in the published studies. The 24-hour proteinuria data were combined for the meta-analysis; we did not include studies that lacked these data. Leflunomide treatment showed a favorable renal function profile, especially for 24-hour proteinuria (mean difference -0.58, 95%CI -0.78~-0.37, *P*<0.01) with significant heterogeneity(*P*<0.00001, I^2^ = 94%) The overall effect was calculated using the fixed effects model and was found to be Z = 5.56 (*P*<0.01), indicating that after leflunomide treatment, 24-hour proteinuria decreased significantly compared with that after cyclophosphamide (Fig 3). Serum creatinine (mean difference -0.20, 95%CI -0.39~-0.01, *P*<0.05) exhibited even better profiles after leflunomide treatment and after cyclophosphamide treatment. Patients receiving cyclophosphamide appeared to have a slightly higher risk of experiencing a transient increase in serum creatinine. Seven studies analyzed serum albumin as an outcome and the leflunomide therapy tended to have better efficacy regarding serum albumin, but this difference was not statistically significant (mean difference -0.02, 95%CI -0.25~0.20, *P* = 0.85) with significant heterogeneity (*P*<0.0001, I^2^ = 79%).

The safety outcomes included adverse events such as liver function abnormality, gastrointestinal reaction, rash, alopecia, leucopenia, infection, menoxenia and herpes zoster infection. These results are presented in Table 2. A fixed effects model was used because the studies were homogeneous for all ADRs. Liver damage was the most common ADR and was compared between the leflunomide and cyclophosphamide groups in nine trials (n = 447). The pooled RR was 0.53 (95%CI = 0.33~0.87), which indicated that leflunomide was significantly superior to cyclophosphamide in reducing the risk of liver damage. Significantly fewer patients who received leflunomide developed alopecia, leukopenia or infection, with RRs of 0.38 (95%CI = 0.17~0.85), 0.25 (95%CI = 0.08~0.77) and 0.54(95%CI = 0.32~0.92), respectively. The risks of the following ADRs were not significantly different between the two groups: gastrointestinal reaction, 0.73 (95%CI = 0.45~1.17); rash, 1.32 (95%CI = 0.54~3.23); menoxenia, 0.25 (95%CI = 0.05~1.15); and herpes zoster infection, 0.33 (95%CI = 0.04~3.12). The RRs favored the leflunomide group for all ADRs, except rash [2.54 (95%CI = 1.70~3.80)] which occurred slightly more frequently in the leflunomide group, but this difference was not statistically significant (*P* = 0.55).

**Table 2:** Meta-analysis of ADRs in LN patients under leflunomide and cyclophosphamide therapy.

ADRs	Included studies	Trials n/N	Control n/N	RR (95% CI)	P value	Heterogeneity
ALT abnormity	9	16/225	32/222	0.53(0.33, 0.87)**	0.01	P = 0.58, I^2^ = 0%
Gastrointesntial Reaction	8	19/198	33/204	0.73(0.45, 1.17)	0.19	P = 0.50, I^2^ = 0%
Rash	7	9/176	6/171	1.32(0.54, 3.23)	0.55	P = 0.70, I^2^ = 0%
Alopecia	7	6/167	21/173	0.38(0.17, 0.85)*	0.02	P = 0.99, I^2^ = 0%
Leukopenia	6	1/134	11/129	0.25(0.08, 0.77)*	0.02	P = 1.00, I^2^ = 0%
Infection	5	11/121	23/121	0.54(0.32, 0.92)*	0.02	P = 0.31, I^2^ = 0%
Menoxenia	4	0/106	6/106	0.25(0.05, 1.15)	0.07	P = 0.99, I^2^ = 0%
Herpes zoster	2	0/65	2/65	0.33(0.04, 3.12)	0.34	P = 1.00, I^2^ = 0%
Total	7	32/187	69/182	0.45(0.31–0.64)**	<0.001	P = 0.91, I^2^ = 0%

Note: n/N, total events / patients of the group

*P≤0.05

**P≤0.01.

**Fig 4:**
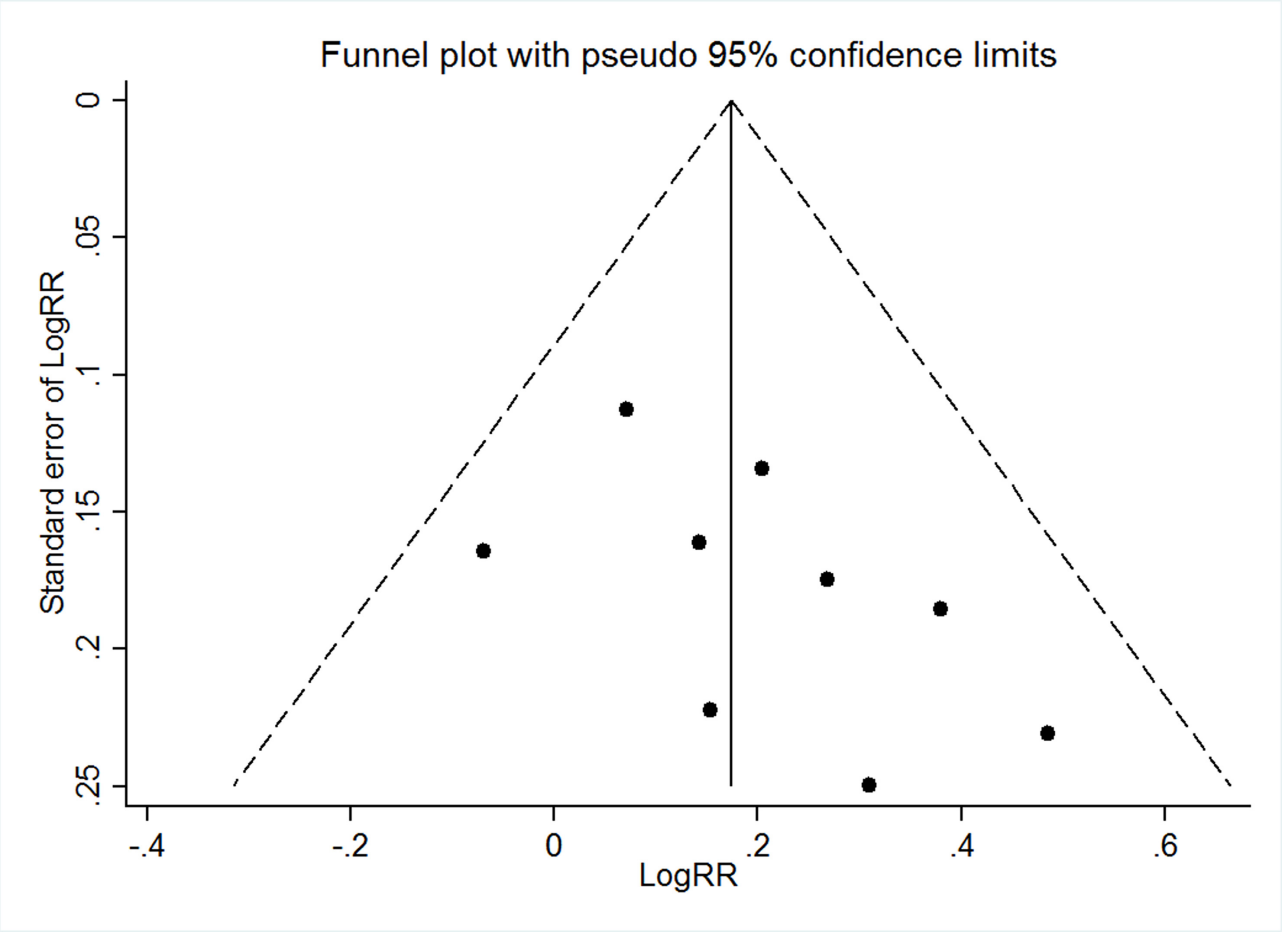
Funnel plot standard error by odds ratio for total remission.

Sensitivity analysis was performed to analyze the high heterogeneity in some study results. The stability results were showed in S4–S6 Tables. These results indicated that the study of Mo[15] et al. may influence the combined results in the outcomes of SLEDAI score and serum albumin. The study of Mo[15] et al. and Peng[17] may influence the combined results in the outcome of 24-hour proteinuria. So we eliminated these studies and performed meta-analysis again (S1 Fig), a random effects model was applied for the meta-analysis. We found the similar results in 24-hour proteinuria (mean difference -0.8, 95%CI -1.38~-0.22, *P*<0.01, I^2^ = 83%) and serum albumin (mean difference 0.92, 95%CI -0.17~2.00, *P*>0.05, I^2^ = 0%). However, we found a different result in SLEDAI score (mean difference -0.34, 95%CI -0.64, -0.04, *P*<0.05, I^2^ = 0%). No evidence of publication bias for the total remission was found in our analysis by a funnel plot (Fig 4) and Egger test (*P* = 0.91).

## Discussion

A meta-analysis is advantageous when a large number of studies can be pooled based on similar criteria. A clinical study addressing such a diverse clinical spectrum as LN would be difficult to design, but the meta-analysis methodology may help elucidate the differences between studies of LN. In the current meta-analysis of 11 RCTs including 254 patients with LN, we summarized the data regarding the efficacy and safety of leflunomide for comparison with those of cyclophosphamide in the treatment of LN. Leflunomide was equivalent to cyclophosphamide in terms of efficacy, but was safer than cyclophosphamide. Leflunomide had similar efficacy compared with cyclophosphamide in terms of the SLEDAI score and serum albumin. In addition, leflunomide was superior to cyclophosphamide in the complete remission rate, total remission rate of lupus nephritis and improvement of renal function. Our meta-analysis generally agrees with previously published RCT reports [10–20] and one published systemic review [7]. There was a significant reduction in ADRs with leflunomide compared with cyclophosphamide. The major advantage of leflunomide is its lower risk for several clinically important adverse effects, such as liver damage, gastrointestinal reactions, alopecia and infection. The reduction in the incidence of ADRs with leflunomide may be attributed to the unique mechanism of action of leflunomide and to the lower total prednisone dose in patients treated with leflunomide compared with those treated with cyclophosphamide.

Leflunomide reduces T cell and B cell proliferation by through inhibiting dihydroorotate dehydrogenase (DHODH), which leads to decreases in DNA and RNA synthesis and cell proliferation[21]. In lupus mouse model, leflunomide restored the suppression of the T cell response to the level observed in healthy mice, suggesting that leflunomide has potential in the treatment of SLE[22]. Bartlett also found that leflunomide reduced the amount of auto-antibodies and immune complex deposits on the glomeruli[23]. He also observed that leflunomide dramatically reduced the production of autoantibodies and immune complex deposition in the kidney, leading to decreased kidney damage and reduced mortality in cGVHD (chronic graft vs host disease) induction [24]. Remer[25] explored the potential efficacy and tolerability of leflunomide in SLE patients who were not adequately controlled by concurrent or previous medications. Tam et al. [26] conducted the first double-blind, randomized, placebo-controlled pilot study of leflunomide in SLE. The SLEDAI score decreased to a greater extent in the leflunomide group than in the placebo group from baseline to 24 weeks. Only minor adverse events were observed, such as transient elevation in Alanine aminotransferase (ALT), hypertension and transient leucopenia. In a prospective multi-center observational study conducted by Wang and her colleges [27], patients with biopsy-proven proliferative LN were assigned to receive the treatment either leflunomide or cyclophosphamide with concomitant prednisone. Renal parameters and the SLEDAI score improved significantly and similarly in both groups. Serum creatinine decreased in both treatment groups. Repeat biopsy for pathological analysis also showed a significant reduction in active lesions in the kidney after 6 months of leflunomide treatment. The major adverse events were similar in both treatment groups. Zhang [2] evaluated the efficacy and safety of leflunomide therapy in LN by repeat kidney biopsy. Repeated kidney biopsy in 31 patients after a full year of leflunomide therapy indicated that 13 patients had transformed from complex types of LN to simple types of LN. The total transformed rate was 41.9%. Leflunomide may be another attractive immunosuppressive drug that could effectively replace traditional immunosuppressant drugs for the treatment of LN if patients become intolerant to other drugs or if other drugs are not effective. But previous studies haven’t found any biomarker or genetic variant to predict the response to leflunomide in LN patients.

This study has several strengths, including the consistency and homogeneity of the design. However, several limitations should also be considered. First, all trials had small sample sizes, and not all studies were double blinded. Additionally, no high quality studies (Jadad score ≥ 5) were identified. The randomization method was adequate in three trials. All trials reported the descriptions of withdrawals. High heterogeneity was observed in some results. The removal of the Mo et al. [15] study from the meta-analysis revealed that this study had contributed to the high heterogeneity in some outcomes such as SLEDAI score and serum albumin, because values for I2 were reduced (I2 = 0%). Though these RCTs were similar in baseline characteristics of patients, there were a few heterogeneities in clinical features, such as drug dosages, pathologic type of LN and different SLEDAI score in baseline. It should be noted that all the studies involved Chinese patients, the efficacy and safety of leflunomide for LN in other race patients need to be proven in further research. Second, most study durations were six months and important outcomes, such as mortality and end-stage renal failure profiles, could not be extracted for comparison. Third, remission and renal function were likely very carefully and systematically measured as primary outcomes, while adverse events were usually reported as secondary endpoints or spontaneously; thus, the strength of evidence regarding ADRs may be low[28]. Additionally, the included trials did not provide complete information regarding all the adverse events, which compromised the conclusions that could be drawn based on the results. Our results should be interpreted with caution. Studies with longer follow-up duration and more patients are required to better assess the efficacy and safety profile of leflunomide in LN.

## Conclusions

Our results demonstrated that leflunomide is a promising therapy for LN treatment, primarily because of the comparable efficacy and preferable safety profile determined by this meta-analysis of RCTs. Leflunomide may be a treatment option for patients with LN who have not responded to cyclophosphamide. However, our results should be interpreted with caution because of the small sample size and high heterogeneity. Larger RCTs with longer duration of observation are necessary to provide strong evidence of the efficacy and safety of leflunomide in LN patients.

## Supporting Information

S1 Fig Supplementary results of the meta-analysis. (TIF)

S1 Table PRISMA checklist. (DOC)

S2 Table Search strategy in Pubmed. (DOC)

S3 Table Summary of available information for excluded studies. (DOC)

S4 Table Sensitivity analysis for 24-hour proteinuria. (DOC)

S5 Table Sensitivity analysis for serum albumin. (DOC)

S6 Table Sensitivity analysis for SLEDAI score. (DOC)
