# Supplementary figures and images for: The Efficacy and Safety of Leflunomide for the Treatment of Lupus Nephritis in Chinese Patients: Systematic Review and Meta-Analysis
Source: PLoS One. 2015 Dec 15;10(12):e0144548. doi: 10.1371/journal.pone.0144548 (PMC4686023; doi:10.1371/journal.pone.0144548)

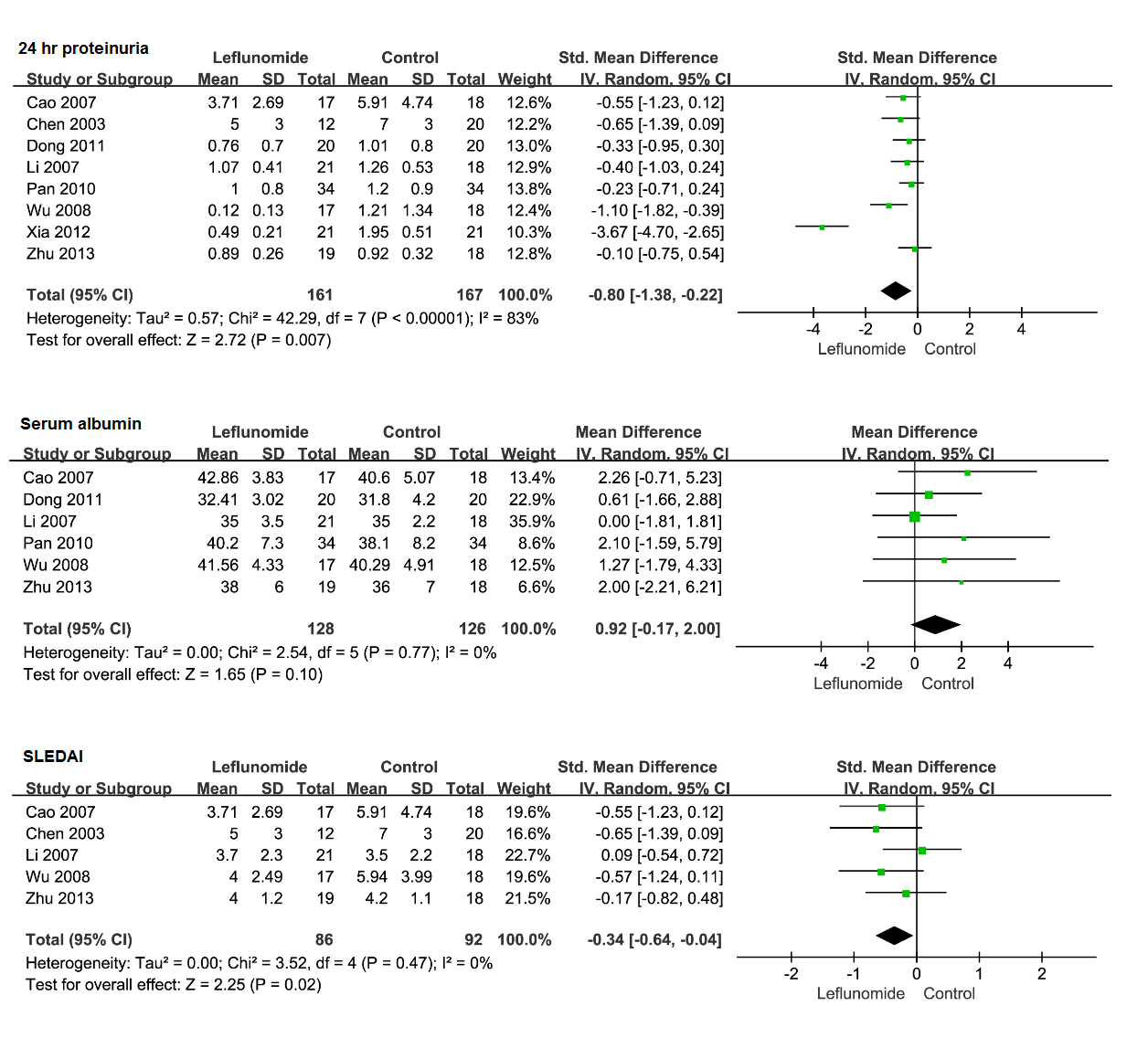

Supplement: S1 Fig — (TIF) [file pone.0144548.s001.tif]
