# Supplementary material for: The Efficacy and Safety of Leflunomide for the Treatment of Lupus Nephritis in Chinese Patients: Systematic Review and Meta-Analysis
Source: PLoS One. 2015 Dec 15;10(12):e0144548. doi: 10.1371/journal.pone.0144548 (PMC4686023; doi:10.1371/journal.pone.0144548)
Supplement: S2 Table — (DOC) [file pone.0144548.s003.doc]

**S2 Table. Search strategy in Pubmed**

| 1 | Leflunomide [Title/Abstract] |
| --- | --- |
| 2 | LEF [Title/Abstract] |
| 3 | efficacy [Title/Abstract] |
| 4 | safety [Title/Abstract] |
| 5 | autoimmunity [Title/Abstract] |
| 6 | autoimmune [Title/Abstract] |
| 7 | systemic lupus erythematosus [Title/Abstract] |
| 8 | SLE [Title/Abstract] |
| 9 | Lupus nephritis [Title/Abstract] |
| 10 | Nephritis [Title/Abstract] |
| 11 | Lupus [Title/Abstract] |
| 12 | leflunomide [Supplementary Concept] |
| 13 | safety [Mesh] |
| 14 | Lupus Erythematosus, Systemic[Mesh] |
| 15 | Lupus nephritis [Mesh] |
| 16 | Nephritis [Mesh] |
| 17 | Trials [Title/Abstract] |
| 18 | Clinical trials [Title/Abstract] |
| 19 | Clinical trials as Topic [Mesh] |
| 20 | 1 OR 2 OR 12 |
| 21 | 5 OR 6 OR 7 OR 8 OR 9 OR 10 OR 11 OR 14 OR 15 OR 16 |
| 22 | 3 OR 4 OR 13 OR 17 OR 18 OR 19 |
| 23 | 20 AND 21 AND 22 |

Search (((((Clinical trials[Title/Abstract]) OR "Clinical Trials as Topic"[Mesh]) OR (((((trials[Title/Abstract]) OR (((efficacy[Title/Abstract]) OR safety[Title/Abstract]) OR "Safety"[Mesh]))) OR Clinical trials[Title/Abstract]) OR "Clinical Trials as Topic"[Mesh]))) AND ((lupus[Title/Abstract]) OR ((("Nephritis"[Mesh]) OR nephritis[Title/Abstract]) OR (((((((autoimmunity[Title/Abstract]) OR autoimmune[Title/Abstract]) OR systemic lupus erythematosus[Title/Abstract]) OR SLE[Title/Abstract]) OR "Lupus Erythematosus, Systemic"[Mesh]) OR lupus nephritis[Title/Abstract]) OR "Lupus Nephritis"[Mesh])))) AND (((Leflunomide[Title/Abstract]) OR LEF[Title/Abstract]) OR "leflunomide" [Supplementary Concept])
