# Supplementary material for: The Efficacy and Safety of Leflunomide for the Treatment of Lupus Nephritis in Chinese Patients: Systematic Review and Meta-Analysis
Source: PLoS One. 2015 Dec 15;10(12):e0144548. doi: 10.1371/journal.pone.0144548 (PMC4686023; doi:10.1371/journal.pone.0144548)
Supplement: S3 Table — (DOC) [file pone.0144548.s004.doc]

**S3 table.** Summary of available information for excluded studies

| **Author (study)** | **Design type** | **No. of patients** | | **Comparison** | **Outcomes** | **Follow-up duration** | **Jadad's score** | **Reason for exclusion** |
| --- | --- | --- | --- | --- | --- | --- | --- | --- |
|  |  | **Test** | **Control** |  |  |  |  |  |
| Tam 2004 | RCT | 6 | 6 | LEF+Pred vs. placebo | SLEDAI, proteinuria, ADR | 6 months | 3 | control(placebo) |
| Cui 2005 | non-RCT | 20 | 16 | LEF+Pred vs. CTX+Pred | CR, PR, NR, proteinuria, SLEDAI, ALB, SCr | 6 months | 1 | Jadad's score＜3 |
| Liu 2008 | non-RCT | 15 | 17 | LEF+Pred vs. CTX+Pred | CR, PR, NR, ADR | 1 year | 1 | Jadad's score＜3 |
| Wang 2008 | non-RCT(CCT) | 70 | 40 | LEF+Pred vs. CTX+Pred | CR, PR, NR, proteinuria, SLEDAI, ALB, SCr, C3, ADR | 6 months | 1 | Jadad's score＜3 |
| Yang 2008 | RCT | 20 | 20 | LEF+Pred vs. CTX+Pred | CR, PR, NR, ADR | 6 months | 2 | Jadad's score＜3 |
| Chen 2010 | RCT | 19 | 18 | LEF+Pred vs. CTX+Pred | CR, PR, NR, ADR | 6 months | 2 | Jadad's score＜3 |
| Feng 2013 | RCT | 15 | 17 | LEF+Pred vs. CTX+Pred | CR, PR, NR, proteinuria, SCr, CRP, ESR, ADR | 6 months | 2 | Jadad's score＜3 |
| Jiang 2013 | RCT | 21 | 21 | LEF+Pred vs. CTX+Pred | CR, PR, NR, SLEDAI, proteinuria, anti-dsDNA, C3, ADR | 6 months | 1 | Jadad's score＜3 |
| Sui 2013 | RCT | 31 | 21 | LEF+Pred vs. CTX+Pred | CR, PR, NR, proteinuria, SCr, CRP, ESR, ADR | 6 months | 1 | Jadad's score＜3 |
| Wu 2013 | RCT | 18 | 18 | LEF+Pred vs. CTX+Pred | CR, PR, NR, proteinuria, SCr, CRP, ESR, ADR | 12 months | 1 | Jadad's score＜3 |
| Li 2014 | RCT | 20 | 20 | LEF+Pred vs. CTX+Pred | CR, PR, NR, proteinuria, SCr, CRP, ESR, ADR | 9 months | 2 | Jadad's score＜3 |

Notes: LEF, leflunomide; CYC, cyclophosphamide; Pred, prednisone; SLEDAI, SLE Disease Activity Index; ALB, albumin; ADR, adverse drug reaction; SCr, serum creatinine; CR, complete remission; PR, partial remission; NR, no remission; C3, complement 3; ANA, antinuclear antibodies; CRP, C reactive protein; ESR, Erythrocyte Sedimentation Rate.
