# Supplementary material for: The Efficacy and Safety of Leflunomide for the Treatment of Lupus Nephritis in Chinese Patients: Systematic Review and Meta-Analysis
Source: PLoS One. 2015 Dec 15;10(12):e0144548. doi: 10.1371/journal.pone.0144548 (PMC4686023; doi:10.1371/journal.pone.0144548)
Supplement: S4 Table — (DOC) [file pone.0144548.s005.doc]

**S4** table. Sensitivity analysis for 24-hour proteinuria

| Study omitted | Estimate | [95%CI] |
| --- | --- | --- |
| Cao 2007 | -0.57 | [-0.78, -0.35] |
| Li 2007 | -0.61 | [-0.83, -0.40] |
| Mo 2010 | -0.86 | [-1.08,-0.64] |
| Pan 2010 | -0.67 | [-0.89,-0.45] |
| Peng 2011 | -0.32 | [-0.53,-0.11] |
| Wu 2008 | -0.54 | [-0.75,-0.33] |
| Xia 2012 | -0.46 | [-0.67,-0.25] |
| Zhu 2013 | -0.65 | [-0.86,-0.43] |
| Chen 2003 | -0.65 | [-0.86,-0.44] |
| Dong 2011 | -0.62 | [-0.84,-0.41] |
| Combined | -0.59 | [-0.79,-0.39] |
