# Supplementary material for: The Efficacy and Safety of Leflunomide for the Treatment of Lupus Nephritis in Chinese Patients: Systematic Review and Meta-Analysis
Source: PLoS One. 2015 Dec 15;10(12):e0144548. doi: 10.1371/journal.pone.0144548 (PMC4686023; doi:10.1371/journal.pone.0144548)
Supplement: S5 Table — (DOC) [file pone.0144548.s006.doc]

**S5 table. Sensitivity analysis for serum albumin**

| Study omitted | Estimate | [95%CI] |
| --- | --- | --- |
| Cao 2007 | -0.09 | [-0.33, 0.15] |
| Li 2007 | -0.02 | [-0.27, 0.22] |
| Wu 2008 | -0.06 | [-0.30, 0.18] |
| Mo 2010 | 0.25 | [0.00, 0.50] |
| Pan 2010 | -0.11 | [-0.36, 0.15] |
| Dong 2011 | -0.05 | [-0.29, 0.19] |
| Zhu 2013 | -0.07 | [-0.31, 0.17] |
| Combined | -0.02 | [-0.25, 0.20] |
