# Supplementary material for: The Efficacy and Safety of Leflunomide for the Treatment of Lupus Nephritis in Chinese Patients: Systematic Review and Meta-Analysis
Source: PLoS One. 2015 Dec 15;10(12):e0144548. doi: 10.1371/journal.pone.0144548 (PMC4686023; doi:10.1371/journal.pone.0144548)
Supplement: S6 Table — (DOC) [file pone.0144548.s007.doc]

**S6 table. Sensitivity analysis for** SLEDAI score

| Study omitted | Estimate | [95%CI] |
| --- | --- | --- |
| Chen 2003 | -0.04 | [-0.31, 0.24] |
| Cao 2007 | -0.04 | [-0.32, 0.24] |
| Li 2007 | -0.16 | [-0.44, 0.13] |
| Wu 2008 | -0.04 | [-0.31, 0.24] |
| Mo 2010 | -0.35 | [-0.65, -0.05] |
| Zhu 2013 | -0.1 | [-0.38, 0.18] |
| Combined | -0.11 | [-0.37, 0.14] |
